# Becoming human in the age of AI: cognitive co-evolutionary processes

**DOI:** 10.3389/fpsyg.2025.1734048

**Published:** 2026-01-14

**Authors:** Anders Högberg

**Affiliations:** 1 School of Cultural Science, Archaeology, Linnaeus University, Kalmar, Sweden; 2 Palaeo-Research Institute, University of Johannesburg, Auckland Park, South Africa

**Keywords:** AI-human cognitive co-evolution, cognitive archeology, cognitive evolution studies/archeology, embodied cognition, evolutionary mismatch, future studies, neuroplasticity, stone age brain

## Abstract

This perspective article brings to focus the unpredictable trajectory of AI-human cognitive co-evolution. Challenging the notion of a fixed ‘Stone Age brain’, it emphasizes the adaptive and plastic nature of human cognition shaped by millions of years of technological engagement. Underlining the need for anticipatory thinking, it asks: What do we need to know now, to be able to recognize what people need to understand in a yet unexplored future of AI-human cognitive co-evolution? Rather than presenting empirical findings, this theoretical and exploratory piece seeks to stimulate reflection and dialog on how AI’s integration into human life may transform our notions of humanness, as AI systems are reshaping human cognition, relationships, and socio-technical practices.

## 1 Introduction

The integration of artificial intelligence (AI) into human life is beginning to reshape our understanding of what it means to be human. This transformation extends beyond individual skills and abilities, prompting deeper questions about the nature of AI-human interaction in futures characterized by profound and seamless AI-human co-operative co-evolutionary processes.

Extensive research provides insights into effects of AI’s use—at individual, organizational, and societal levels (Carroll et al., 2024). Some scholars are highly optimistic (Rawas, 2024), envisioning futures that yet only exist in science fiction (see discussion in Cave and Dihal, 2019). Others express skepticism and view AI as an existential threat (van der Gun and Guest, 2024; Kasirzadeh, 2025), foreboding the end of the *c*. three-million-year-old evolutionary *Homo* lineages´ on Earth (Carlsmith, 2024). Yet, others place themselves somewhere in between these positions (see discussion in Farahani and Ghasemi, 2024; Larsson and Viktorelius, 2024; Qian et al., 2024; Valenzuela et al., 2024; Guingrich and Graziano, 2025; Na and Zhang, 2025).

Here I take another approach. Drawing on Embodied Cognition, Cognitive Evolution Studies/Archeology, and Futures Studies, I start from the question:

- What do we need to know now, to be able to recognize what people need to understand in a yet unexplored future of AI-human cognitive co-evolution?

My core argument is that the same adaptive capacities that enabled *Homo sapiens* to thrive in diverse environments on all continents, will also shape our co-evolution with AI. And, as our modes of engagement co-evolve with AI, so too do our conceptions of the human condition and our experiences of what it means to be human. Hence, we have entered an AI-human cognitive co-evolutionary trajectory whose outcomes are unpredictable (Raman et al., 2025). Few have however addressed what this implies for our understanding of what it means to become human in the age of AI (Andres, 2025; Rainey and Hochberg, 2025). For the sake of my arguments, I will here assume that our use of AI technologies will evolve in ways that currently lie beyond our capacity to foresee. In doing so, I ascribe neither positive nor negative connotations to this development (see Guest et al., 2025a for discussion). Although I use the term AI broadly, I am primarily referring to technologies expected to evolve from what we currently understand as Artificial General Intelligence (AGI). AGI is generally defined as AI systems capable of integrating cognitive, emotional, and reasoning abilities across multiple domains to “understand, learn, and adapt to perform any intellectual task a human can perform” (Raman et al., 2025, p. 3). While the timeline for AGI implementation remains uncertain, it is likely to occur in the near future (Yenduri et al., 2025).

## 2 Technological engagement, a defining characteristic of *Homo sapiens*

Humans’ dependence on tools and technologies is not only a universal feature across cultures but also a fundamental aspect of our evolutionary history (Lombard et al., 2021). Over millions of years, the human socio-technical niche has co-evolved with our cognition through technological engagement, intricately embedded within socio-cultural contexts, our body and the environment we live in. As Lombard et al. (2021), p. 144) show, the way we understand or think about the world individual and collective extended cognition (Clark and Chalmers, 1998; Candiotto, 2023), is continuously being shaped and re-shaped in feedback loops by our technologies, our biology, social interaction with others and our ecological niche/s (Andres, 2022).

The earliest known evidence of this co-evolution is represented by stone tools dating back approximately 3.3 million years (Lombard et al., 2021). This means that our very survival is inseparably linked to technological intervention. We are all obligatory tool users and cannot exist without tools, as individuals or society (Shea, 2017). Hence, technologies are not addons to our experiences of what it means to be human; they are constitutive of it (Gärdenfors and Högberg, 2017; Riede et al., 2018; Crombé, 2019; Frieman, 2021; Lombard et al., 2021; Högberg et al., 2024).

Along these lines, Malafouris (2021, p. 38) explains that humans are inextricably intertwined with the plasticity of forms that we make. Technological performances and practices “alter the ecology of our minds, re-configure the boundaries of our thinking and the ways we make sense of the world” (Malafouris, 2015, p. 351). The blind man’s stick is a well-known example illustrating how cognition extends into tools through embodied interaction (Malafouris, 2013). The tip of the stick is not merely an external aid—it becomes part of the perceptual system, allowing the user to ‘hear, feel and think’ the environment integrated as part of the body. Through continuous sensorimotor coupling, the stick integrates with the hand and arm, transforming spatial awareness and guiding action. This demonstrates that thinking and perceiving emerge from the dynamic interplay of brain, body, material artifacts and environment.

Hence, technologies are not passive instruments in human hands; they are key factors and agents—or actants, in Latourian terms (Latour, 2007)—in shaping human cognition (White et al., 2025). Along similar lines, Federico et al. (2025) situate what they term ‘technological cognition’ within the broader concepts of embodied and extended cognition, suggesting that technology expand human mental capacities and actively influence not only our cognition but also the structure and functioning of the mind itself (see also Osiurak and Reynaud, 2019; Osiurak et al., 2025).

Thus, the human mind, defined by its neuroplasticity (Lombard et al., 2021; Diniz and Crestani, 2023), is dynamically co-constituted through its embodied interaction with technologies (Lombard, 2025a, 2025b). This has not only shaped our deep evolutionary past but continues to influence us today (Solms and Turnbull, 2002). Consequently, it will remain a formative force in our engagement with AI technologies, as they influence and change our socio-cultural contexts, our bodies and the environments we live in (Andres et al., 2025 and Raman et al., 2025).

## 3 The myth of the ‘stone age brain’ and human capacity for adaptation

Over the years, numerous writers in academic and popular science contexts have argued that we possess a ‘Stone Age brain’ ill-suited to modern society (e.g., Hansen, 2019; Kenrick and Lundberg-Kenrick, 2022; Cytowic, 2024). This line of reasoning has deep epistemological roots, echoing millennia-old philosophical debates about humanity’s ‘state of nature’ as a normative guide for behavior (see Høiris, 2016). The central argument in contemporary discussions is that our brains were shaped by ancestral environments and have remained largely unchanged since prehistory. According to this line of reasoning, we are cognitively programmed for the demands of a prehistoric hunter-gatherer reality—vastly different from the modern experiences encountered in the WEIRD parts of the world (WEIRD = Western, Educated, Industrialized, Rich, and Democratic; see Henrich et al., 2010 for discussion).

‘Evolutionary mismatch’ has been termed as a core concept to explain our Stone Age brain out of sync with contemporary life (Li et al., 2018). However, the concept of evolutionary mismatch carries with it underlying assumptions that misleads us in our thinking. As Sapolsky (2017) demonstrates, it fosters a false impression of the brain as predetermined for a fixed hunter-gatherer socio-technical *status quo*.

What truly defines human cognitive evolution is our extraordinary capacity for adaptation. Today, humanity thrives across all continents and within a wide range of biomes. We have adapted to inhabiting environments that range from arid, scorching deserts to snow-covered, permafrost-dominated landscapes. Hence, we are simply not only ‘pre-programmed’ for a Stone Age existence, but also well-equipped for variation and change (Lombard et al., 2021; Zeller et al., 2023; Jakobsson et al., 2025).

This is demonstrated in recent advances in cognitive archeology, which uncover the complex structures underpinning human interaction with technology through time (Henley et al., 2020). Lombard (2025a,b), for instance, investigates the neuro-genetic and behavioral foundations of this interaction, focusing particularly on attention. She argues that the evolution of complex techno-behaviors, such as bowhunting, demanded a unique configuration of attentional capacities, especially sustained and visuospatial attention (see also Gärdenfors and Lombard, 2025). By using genetic and archeological data, Lombard shows that *Homo sapiens* evolved distinct brain structures, enhancing our ability to focus, shift, and divide attention across space and time (see also Jakobsson et al., 2025). Lombard (2025a,b) concludes that these capacities may have developed strongly in the precuneus from around 160,000 years ago, reaching modern human ranges by approximately 100,000 years ago, i.e., some 200,000 years after the earliest evidence we have of anatomically modern humans (Schlebusch et al., 2017). This demonstrates that our *Homo sapiens* brain has evolved over time.

Other scholars have explored more rapid processes. It is well established that epigenetics influences how genes are expressed in response to environmental stimuli (Ashe et al., 2021). Rather than altering the DNA sequence, epigenetic mechanisms regulate gene activity, potentially affecting brain development and cognition. These changes may be inherited across generations, providing a dynamic layer of biological plasticity that integrates with the neuroplasticity of the human mind and its embodied interaction with technology (González-Rodríguez et al., 2023).

So, before we move on to the next part of the text, it is important to conclude that human cognition has always evolved through embodied interaction with environmental and socio-technical domains, reinforced by epigenetic processes. We are equipped with a neuroplastic brain primed to seamlessly co-evolve with technology. We are not simply ‘pre-set’ with a Stone Age mind, our cognitive evolution is continuous and ongoing. Our evolutionary history gives us exceptional abilities to adapt to variation and change. As a result, we are perfectly primed to cognitively co-evolve with AI, as AI systems become integrated into our cognition.

## 4 AI and the reshaping of human cognition

Today, AI is an integral part of human life, shaping activities such as communication, entertainment, transport, healthcare, and education (Rainey and Hochberg, 2025). It is also beginning to influence the plasticity of human cognition as it becomes increasingly integrated into both brain and body (Andres, 2025; Raman et al., 2025). AI can, for instance, assist individuals who have lost the ability to speak in partially regaining it (Littlejohn et al., 2025), enable blind people to develop a unique perception of the world (Tang, 2025), and replace lost limbs with AI-powered neural devices (Lee et al., 2025). Moreover, as AI systems interact with humans, feedback loops begin to influence cognition. Hohenstein et al. (2023), Andres (2025) and Glickman and Sharot (2025), for example, have demonstrated how AI-human interactions alter cognitive processes underpinning communication and social relationships as well as human agency, responsibility and care, and moreover perceptual, emotional, and social judgments (see also Pedreschi et al., 2025).

By using the complexity of the human brain as a proxy (see discussion in Guest et al., 2025b), researchers have developed deep neural networks to build AI systems. Indeed, the tendency to draw direct comparisons between AI and the human brain is deeply embedded in the history of the field (Durt, 2022, p. 69). And, as human cognition serves as a foundation for building AI, many assume that AI will eventually be able to replicate human thought processes (see discussion in van Rooij et al., 2024). Yet, as Durt (2022, p. 68–69) points out, this AI-human brain analogy has caused much confusion and is “probably the strongest reason for the typical conceptions of AI as a replication or simulation of human intelligence.” Such misinterpretation significantly constrains our ability to envision alternative futures for AI-human co-evolution.

Human cognition is, in many respects, uncertain and irrational (Gärdenfors, 2024). Hence, there is little reason to think that any future AI system (yet unknown to us), whether capable of simulating agency or genuinely possessing it, would seek to replicate human cognition in all its flawed complexity. Rather, we must recognize the distinct and different evolutionary paths of humans and AI.

Human cognition has evolved over millions of years through complex interactions between biology, society, ecology, and technology. It is deeply interwoven with episodic memory, our exceptional capacities for learning and teaching, empathy, theory of mind, and our advanced abilities to narrate and comprehend multiple spatio-temporal intentions and consequences, just to name a few examples (Tomasello, 1999; Donald, 2001; Gärdenfors, 2006; Gärdenfors and Högberg, 2017; Lombard et al., 2021).

By contrast, AI is developing over a relatively short time frame in an environment where emotions and morality can be simulated but are not physically experienced and embodied. It possesses the capacity to learn from vast datasets. The systems we currently interact with are global in scale, trained on data equivalent to thousands upon thousands of human lifetimes. These systems can transfer information directly between one another, unconstrained by the physical and emotional limits that characterize human cognition (AI-2027, n.d.).

## 5 Augmented cognition

Siemens et al. (2022, p. 6) argue that augmented cognition, i.e., the use of real-time data analysis and adaptive systems to enhance cognitive processes, “supports information processing related to sensory memory, working memory, executive functions, and attention,” and further “provides access to an extended memory and virtually unlimited knowledge.” Such augmentation fosters synergies that enhance decision-making, problem-solving, and creative capacities by leveraging the strengths of both human cognition and machine precision. These emergent capabilities arise from the dynamic interplay between humans and AI, resulting in abilities that neither humans nor AI could achieve independently (Pedreschi et al., 2025).

It is therefore reasonable to anticipate that AI-human interaction will give rise to new forms of augmented cognition, potentially expanding our current understanding of what cognition entails. Given that the evolutionary trajectories of human and AI cognition differ significantly, it is likely that AI will develop distinctive forms of cognition, very different to humans’ (Raman et al., 2025). Whether such developments will ultimately be recognized as ‘cognition’ remains uncertain. And the pace and nature of AI’s evolution will be fast and so different from what we know from research on human and animal cognitive evolution (e.g., Donald, 2001; de Waal, 2017; Reber, 2024), that we will struggle to understand it (Rainey and Hochberg, 2025).

Consequently, humanity will encounter a form of cognition previously unknown to us. Not since the interbreeding events between *Homo sapiens* and Neanderthals or Denisovans some 45,000 to 65,000 years ago (Iasi et al., 2024; Ongaro and Huerta-Sanchez, 2024) have humans encountered a previous unknown intelligence capable of mimicking/representing human cognition. This new cognition will have super-human abilities. At the same time, it will lack basic human competences, yet mimic and emulate them in ways that create illusions of possessing them [see discussion in Carroll et al. (2024)].

If we accept these premises, it becomes essential to adopt a forward-looking approach to develop new knowledge about what this will mean for how we understand futures with human and artificial cognitive co-evolution.

## 6 Cognitive co-evolution in futures not yet understood

Alford (2025, p. 5) defines ´futures thinking´ as a “cognitive approach and mindset that involves the consideration and exploration of a range of futures and their implications, engaging with complex, long-term perspectives to […] prepare for various possibilities.” It is a systematic way to anticipate and imagine alternative futures and to inform actions in the present. It involves capacities to envision multiple possible futures, diverging from the present. From this perspective, the future should not be conceptualized as a territory to map and conquer, but rather as a generative source of emergent possibilities for present action (Poli, 2021, p. 5).

It is important to emphasize that this is not about forecasting or planning for a particular future to materialize, as is often the case in for example current debates surrounding utopian and dystopias AI-futures [see discussion in Carroll et al. (2024)]. Rather, the focus lies in cultivating our foresight capacities: the ability to imagine a plurality of futures that diverge significantly from the present (Holtorf and Högberg, 2021). This process entails, among other things, becoming critically aware of the assumptions we currently hold, and recognizing how these may constrain our ability to explore AI-human co-evolution in novel ways (Pauketat et al., 2025).

As discussed by Raman et al. (2025), the future of AI-human co-evolution will be another reality, characterized by new modes of being, doing, living, and knowing that differs from those of both the present and the past (see also Andres, 2022). Hence, future generations will confront challenges within their own contemporary contexts that we, in the present, cannot foresee. They will inevitably engage with their world through their own frameworks of understanding, shaped by what resonates with their lived experiences (Jae, 2023). In doing so, they will reinterpret their past (which is our present) stripping it of the meanings we now consider significant, to equip themselves with the knowledge necessary to act within their own time (which for us remains futures yet to unfold). It challenges us to ask questions like:

- How might AI-human collaboration redefine future understandings of what it means to be human?
- What new forms of knowledge are required to identify uniquely human skills and abilities in such futures?
- How might these skills and abilities develop in co-evolutionary processes, as AI systems emulate human cognition and develop what might be perceived as cognition?

Moreover, a significant challenge lies in the fact that future generations will differ from one another, necessitating distinct approaches tailored to their respective contexts. Hence, the task at hand is not a matter of translating into a single, unknown future, but rather of engaging in multiple re-translations across a succession of future contexts, unknown in advance (Terry et al., 2024; Holzheu et al., 2025). To begin exploring this terrain, we must first become adept at recognizing the emerging capacities within AI-human co-evolution even before we fully comprehend what we are seeking. This requires us to reflect on what the very act of understanding will entail, as cognition itself evolves and becomes seamlessly distributed across humans and AI (Table 1).

**Table 1:** Challenges and questions.

Challenges	Questions
Cognitive work redistribution	What aspects of deeper thinking will become weakened or strengthened in humans and/or in AI? How can people become skilled in recognizing when they have externalized something that perhaps ought to remain internal? What impact will cognitive offloading have on creative and critical thinking?
Skills for recognizing future AI interaction patterns	What questions have we stopped asking ourselves? Are we cultivating new cognitive strengths, or merely developing new dependencies for future generations?
Second-order effects	What new cognitive demands arise in future AI-augmented environments? How can agency be maintained and/or developed in futures when it is no longer possible to reconstruct the chain of reasoning behind decision-making processes?
Futures with alternative options	What future AI-human co-evolution capabilities might emerge, that we currently lack the vocabulary to conceptualize? What new forms of future cognition may become possible?

The edited results of interacting with AI (LLM Claude) to brainstorm on the question that this text starts from: What do we need to know now, to be able to recognize what people need to understand in a yet unexplored future of AI-human cognitive co-evolution?. It is provided here as a ‘meta-example’ of augmented cognition (see S1 for details).

In this lies a paradox: the difficulties inherent in trying to understand futures our current cognitive setup may be inadequate to even perceive correctly. Beginning to explore ways to overcome this paradox opens a research field we have only recently begun to recognize. It may help us understand how we can prepare ourselves to anticipate the kinds of knowledge and insight people will need in a yet-to-be-charted future shaped by the co-evolution of human and artificial cognition.

## Data Availability

The original contributions presented in the study are included in the article/Supplementary material, further inquiries can be directed to the corresponding author.
